# Basophil Activation Test With Aspergillus Molecules: The Case for ABPA

**DOI:** 10.3389/falgy.2022.898731

**Published:** 2022-06-22

**Authors:** Moïse Michel, Youssouf Sereme, Farid Mankouri, Marion Gouitaa, Clarisse Gautier, Jean-Louis Mège, Carole Cassagne, Stéphane Ranque, Martine Reynaud-Gaubert, Joana Vitte

**Affiliations:** ^1^Laboratoire d'Immunologie, CHU Carémeau Nîmes, Nîmes, France; ^2^Aix-Marseille Université, APHM, IRD, MEPHI, Marseille, France; ^3^Assistance Publique—Hôpitaux de Marseille, Marseille, France; ^4^Aix-Marseille Université, APHM, IRD, VITROME, Marseille, France; ^5^IDESP, INSERM UA11, Université de Montpellier, Montpellier, France

**Keywords:** allergic bronchopulmonary aspergillosis (ABPA), basophil activating test (BAT), *Aspergillus fumigatus*, aspergillus molecular allergens, *ex vivo* technique

## Abstract

**Background:**

Allergic bronchopulmonary aspergillosis (ABPA) is an underestimated allergic disease due to *Aspergillus fumigatus* (AF). The main diagnostic criteria for ABPA rely on the evaluation of immunoglobulin (Ig) E and IgG responses to AF extracts, although these cannot discriminate AF-sensitization from ABPA.

**Objectives:**

To evaluate the performance of cellular functional assays with extract and molecular AF allergens in ABPA.

**Methods:**

A prospective cohort of 67 patients (6 ABPA) was investigated with basophil activation test (BAT) with AF extract. Twelve patients were further investigated for BAT responses to molecular AF components: Asp f 1, Asp f 2, Asp f 3, Asp f 4, and Asp f 6.

**Results:**

BAT with AF extract with an optimized cutoff displayed 100% sensitivity and 77.6% specificity for ABPA diagnosis. Among patients with positive BAT to AF, BAT with Asp f 4 was significantly higher in ABPA patients at 10 ng/mL (mean basophil stimulation index 10.56 in ABPA vs. 1.24 in non-ABPA patients, *p* = 0.0002).

**Conclusion:**

BAT with AF is a promising diagnostic biomarker in the context of suspected ABPA, which can be further improved with AF molecular allergens, especially Asp f 4.

## Introduction

Allergic bronchopulmonary aspergillosis (ABPA) is an underestimated allergic disease due to the ubiquitous mold *Aspergillus fumigatus* (AF). ABPA occurs mainly in patients with a chronic pulmonary disease, such as cystic fibrosis (CF), asthma or chronic obstructive pulmonary disease (COPD) (1). Its prevalence reaches 10 and 2% in CF and asthma patients, respectively (2, 3). Up to now, the reason why some AF-sensitized people stay free of subsequent AF-related disease whereas others develop ABPA with irreversible pulmonary lesions remains unknown. The main diagnostic criteria for ABPA, first established in 1977 (4), rely on the evaluation of humoral IgE and IgG responses to AF extracts, which cannot discriminate ABPA from AF-sensitization. Several new diagnostic tools have been evaluated, but none has overcome this limitation (2, 5, 6). More recently, serum immunoglobulin (Ig) E responses to AF molecular components have been proposed, and the combination of specific AF molecular components showed promise for ABPA diagnosis (7–9). Meanwhile, diagnostic criteria based on the evaluation of functional cellular responses against allergens are increasingly cited in international guidelines (10, 11). Performance of the basophil activation test (BAT) with AF extract has been explored (12–16), but its relevance for ABPA diagnosis needs further evaluation. To our best knowledge, AF-induced basophil reactivity has not been deciphered at a molecular level, despite promising results of profiling IgE responses to such molecules. We report here the performance of BAT with AF extract and AF molecular allergens vs. usual diagnostic criteria of ABPA in a prospective cohort.

## patients and Methods

### Patient Cohort

We assessed adult patients (*n* = 67) followed at the CF reference care center and at the pulmonology department (Assistance Publique—Hôpitaux de Marseille, France) between January and September 2019. Patients were categorized as ABPA, AF-sensitized (AF-S), or control patients. These categories were defined as follows: ABPA met all the ISHAM criteria (2); AF-S displayed specific IgE (sIgE) to AF (0.1 kUA/L or greater) without fulfilling the ISHAM criteria for ABPA; and patients who were categorized in none of the two previous groups were considered as controls. According to ISHAM criteria, ABPA is made in patients with a lung predisposing condition who display AF sensitization and elevated total IgE levels and at least 2 of the following 3 criteria: (1) detection of AF specific IgG, (2) radiographic abnormalities, (3) total eosinophil count above 500 cells/μL.

### Functional Cytometric Tests

**Figure 1:**
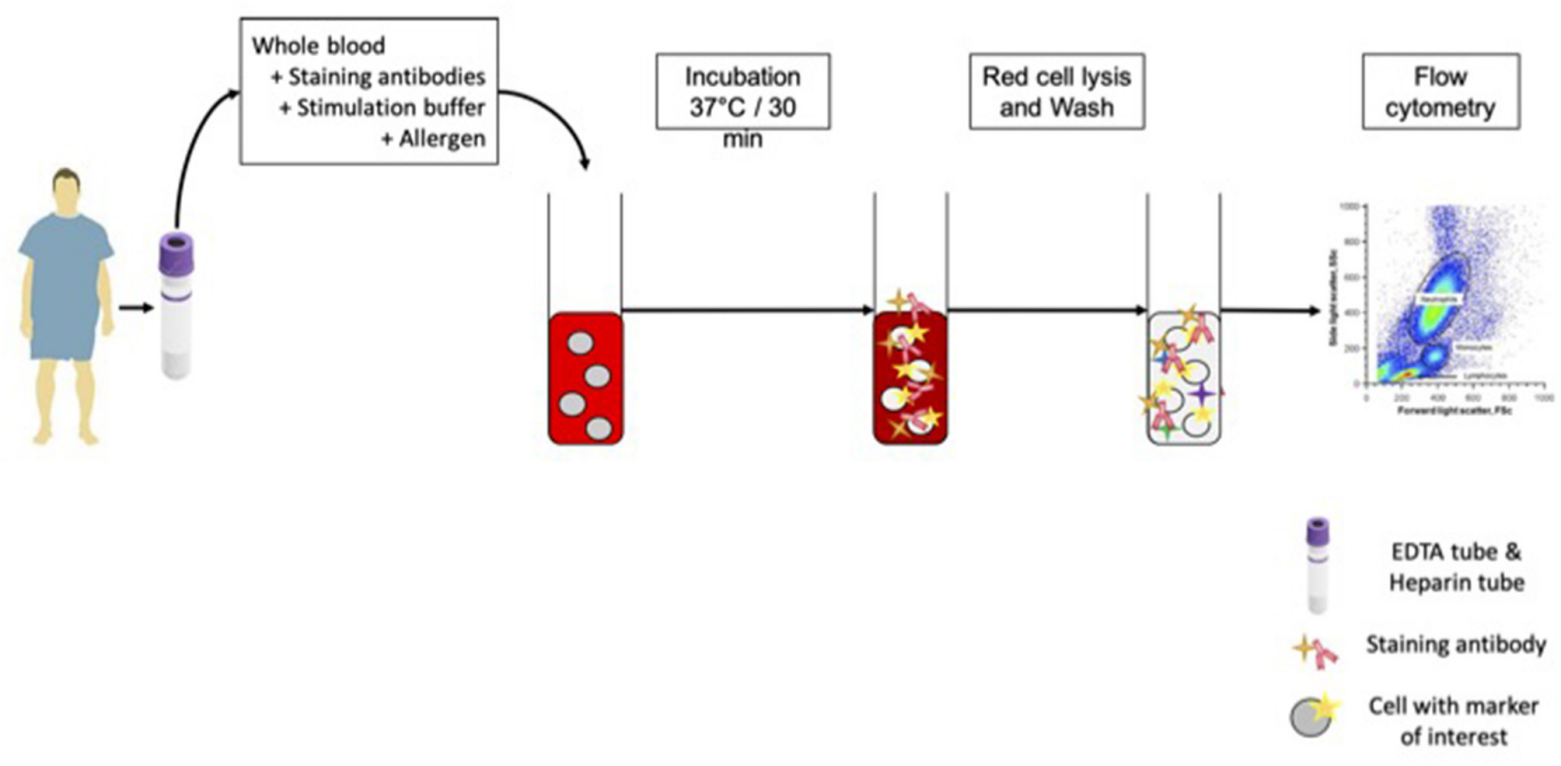
BAT protocol. For each patient in the study, we sampled 1 EDTA tube for BAT. The whole blood was mixed with staining antibodies, stimulation buffer and allergen or controls. After incubation at 37°C, red cell lysis and wash, cells were analyzed on FACS.

The design of functional cellular assays is illustrated in Figure 1. BAT was firstly performed with AF extract (Bühlmann Laboratories®, Schönenbuch, Switzerland) with the Flow2CAST method (Bühlmann Laboratories®), using CCR3 (CD193) and CD63 as basophil identification and activation markers, following the manufacturer's instructions (17). Upon allergen contact, sensitized basophils degranulate and express CD63 as a cell surface activation marker, measurable by flow cytometry. For *in vitro* diagnosis, allergen-induced basophil activation is defined as a proportion of CD63+ basophils at least twice higher with the culprit allergen than with the reaction buffer. Positive controls were anti-RFcεI and the bacterial peptide fMLP. Fresh whole blood was incubated with AF extract or controls and staining antibodies for 30 mins at 37°C. After red cell lysis and washes, basophil responses were analyzed by flow cytometry. The proportion of CD63+ unstimulated basophils was lower than 5% in all patients, and no non-responder (anti-FcεRI-induced CD63 expression of 10% or less) was found in this cohort.

Samples with a positive BAT response to AF extract were subsequently assayed with BAT using each of the five following molecular AF: Asp f 1, Asp f 2, Asp f 3, Asp f 4 and Asp f 6 (a gift from Dr Jonas Lidholm, Thermo Fisher Scientific, R&D, Uppsala, Sweden). These molecular AF are the same used in the ImmunoCAP platform. Five concentrations of molecular components from 100 to 0.01 ng/mL by 10-fold dilution were used for each patient.

Flow cytometry was performed with a FACS Canto II platform (Becton Dickinson, Le Pont de Claix, France). At least 200 basophils per sample were acquired for BAT. Data were analyzed using FACS Diva software (TreeStar, Ashland, OR).

### Clinical and Laboratory Data

White blood cell count (Sysmex, Villepinte, France) and serum total IgE and specific IgE (sIgE) and IgG (sIgG) to AF extract and molecular components Asp f 1, Asp f 2, Asp f 3, Asp f 4 and Asp f 6 (ImmunoCAP, Thermo Fisher Scientific, Uppsala, Sweden) were part of routine investigations. The quantification thresholds were 0.10 kUA/L for sIgE and 0.01 mgA/L for sIgG (18, 19). The patient's pulmonary function test results were retrieved from medical charts.

### Data Analysis

Results were expressed as the basophil or lymphocyte stimulation index (SI), which is the ratio between the level of activation with the allergen and the level of activation with reaction buffer, with a threshold of 2 for allergy diagnosis. Statistical analysis was performed with the R statistical software (20). For BAT, optimal cutoff points were determined with the “OptimalCutpoints” package (21). The Youden index, which defines the maximum potential effectiveness of a biomarker to classify a disease status at a specific cutoff, was also calculated (22). Intertest correlation was estimated using Pearson's correlation coefficient. Mean SI of each group were compared *via* Wilcoxon's or Kruskal-Wallis tests as appropriate. A two-sided *p*-value < 0.05 was considered statistically significant.

### Ethics Statement

All the experimental protocols were approved by the institutional ethics committee and GDPR commission with the reference number 2019–270. All methods used were carried out in accordance with relevant national guidelines. Written informed consent for participation was obtained for this study in accordance with the national legislation and the institutional requirements (23, 24).

## Results

### Demography

The cohort was comprised of 20 CF patients, 25 asthmatic patients, 4 COPD and 18 patients with other chronic pulmonary diseases (6 pulmonary arterial hypertension, 4 idiopathic pulmonary fibrosis, 3 emphysema, 2 chronic bronchiectasis, 1 lymphangioleiomyomatosis, 1 idiopathic chronic eosinophilic pneumonia, 1 infectious pneumonitis). The ABPA group was composed of 6 patients, most of them were CF patients (4/6). The AF-S group included 25 patients, especially CF (10) and asthmatic (8) patients. The control group (*n* = 36) was composed of 7 CF patients, 15 asthmatic patients, 2 COPD and 12 patients with other chronic pulmonary diseases. Demographic data are summarized in Table 1.

**Table 1:** Demographic and laboratory data of the study cohort.

***(median +/−5–95 percentile)***	**ABPA**	**AF-S**	**Control**	**Total**	***p***
n	6	25	36	67	
Age (years)	37.5 (16.3–61.8)	49.5 (25.0–75.0)	57.0 (26.7–78.0)	53.0 (24.3–76.4)	0.025
Male/Female	3/3	9/16	14/22	27/40	0.797
Cystic fibrosis	4	10	7	20	0.020
Asthma	1	8	15	25	0.514
COPD	1	2	2	4	0.509
Others	0	5	12	18	0.097
Lung transplantation	2/6	6/25	14/36	22/67	0.454
Time since transplantation (years)	8.6 (8.2–9.1)	7.7 (0.6–23.6)	1.7 (0.1–13.2)	4.5 (0.1–19.7)	0.294
Bacterial colonization	3/6	9/25	5/36	17/67	0.019
Fungal colonization	3/6	6/25	2/36	11/67	0.005
Total IgE (kIU/L)	1,132.0 (198.7–5,685.8)	197.0 (16.6–1,139.0)	27.5 (2.0–266.3)	66.7 (3.0–1,413.5)	<10^−3^
IgE AF (kUA/L)	16.8 (0.5–66.3)	0.4 (0.1–15.8)	0.05 (0.01–0.09)	0.10 (0.01–19.9)	<10^−3^
IgG AF (mgA/L)	46.8 (22.0–79.1)	18.2 (4.9–52.9)	12.3 (3.1–56.6)	16.6 (3.4–59.0)	0.002
Eosinophils (/mm^3^)	300 (0–800)	100 (100–600)	100 (0–600)	100 (0–700)	0.554

*ABPA, allergic bronchopulmonary aspergillosis; AF, Aspergillus fumigatus; AF-S, Aspergillus fumigatus sensitization; COPD, chronic obstructive pulmonary disease*.

### BAT With AF Extract Displayed Good Performances for ABPA Diagnosis

**Figure 2:**
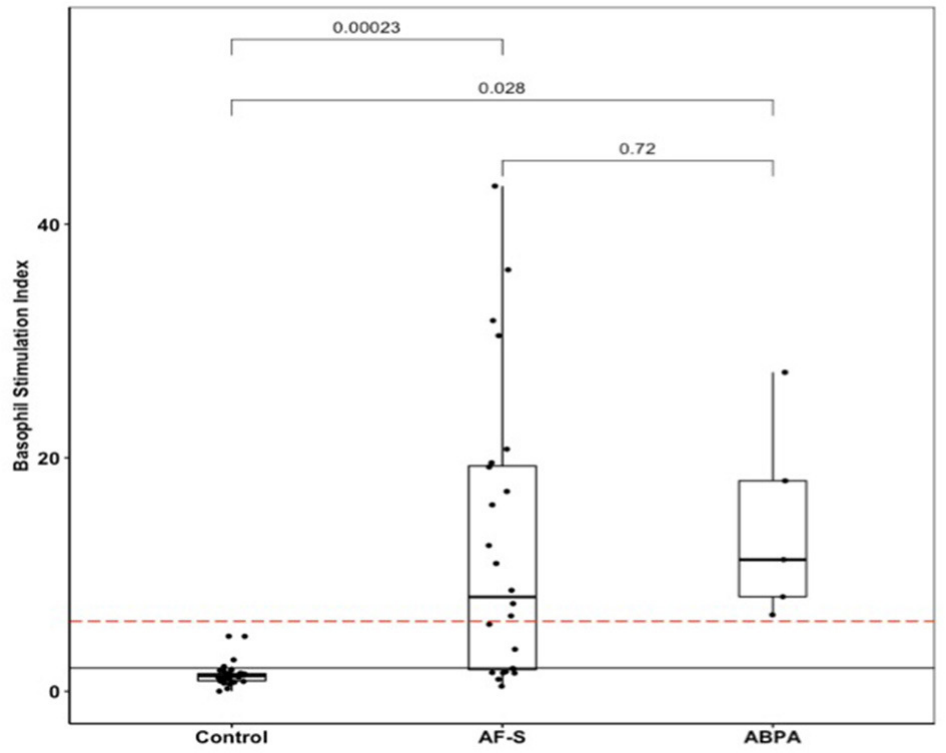
BAT AF in ABPA, AF-sensitized (AF-S), or control patients. The solid black line shows the usual BAT positivity threshold (stimulation index = 2); the dotted red line shows the optimal BAT threshold (stimulation index = 6.55). ABPA, allergic bronchopulmonary aspergillosis; AF, Aspergillus fumigatus; AF-S, Aspergillus fumigatus-sensitized; BAT, basophil activation test.

BAT dose-response to AF extract showed that 50 ng/mL yielded a maximal response, and was therefore the optimal concentration to be used in further BAT (Supplementary Figure 1). BAT was positive in all ABPA patients (Figure 2). BAT discriminated both ABPA (*p* = 0.0028) and AF-S patients (*p* = 0.00023) from controls. However, BAT could not distinguish between ABPA and AF-S patients (*p* = 0.72). Comparing results in ABPA and the control groups, with the usual SI threshold of 2, BAT with AF extract displayed 100% sensitivity and 65.5% specificity. An optimized SI threshold allowing for the best Youden index was calculated as 6.55. This optimized SI retained 100% sensitivity but specificity increased to 77.6% (Table 2). The area under the curve (AUC) was 0.84 BAT with AF extract and 0.83 for sIgE to AF. Youden index was 0.78 for BAT with the optimized SI cut-off, but only 0.66 for sIgE to AF extract.

**Table 2:** Performance analysis of total and specific immunoglobulins and cellular functional assays with the optimized cutoff.

	**Optimized cutoff**	**AUC** **(5–95 percentile)**	**Sensitivity** **(%)**	**Specificity** **(%)**	**PPV** **(%)**	**NPV** **(%)**	**Youden index**
Total IgE	90	0.68 (0.54–0.82)	66.7	66.7	59.3	73.3	0.33
IgE AF	0.24	0.83 (0.71–0.94)	75.0	90.9	85.7	83.3	0.66
IgG AF	27	0.69 (0.53–0.84)	76.2	65.5	61.5	79.2	0.42
BAT with AF extract	6.55	0.84 (0.74–0.94)	100	77.6	27.8	100	0.78

*Optimization consisted in the identification of the cut-off value associated with the best Youden index*.

*AF, Aspergillus fumigatus; AUC, Area under curve; BAT, Basophil activation test; NPP, Negative predictive value; PPV, Positive predictive value*.

### BAT With Molecular Components Further Improved BAT Performance

**Figure 3:**
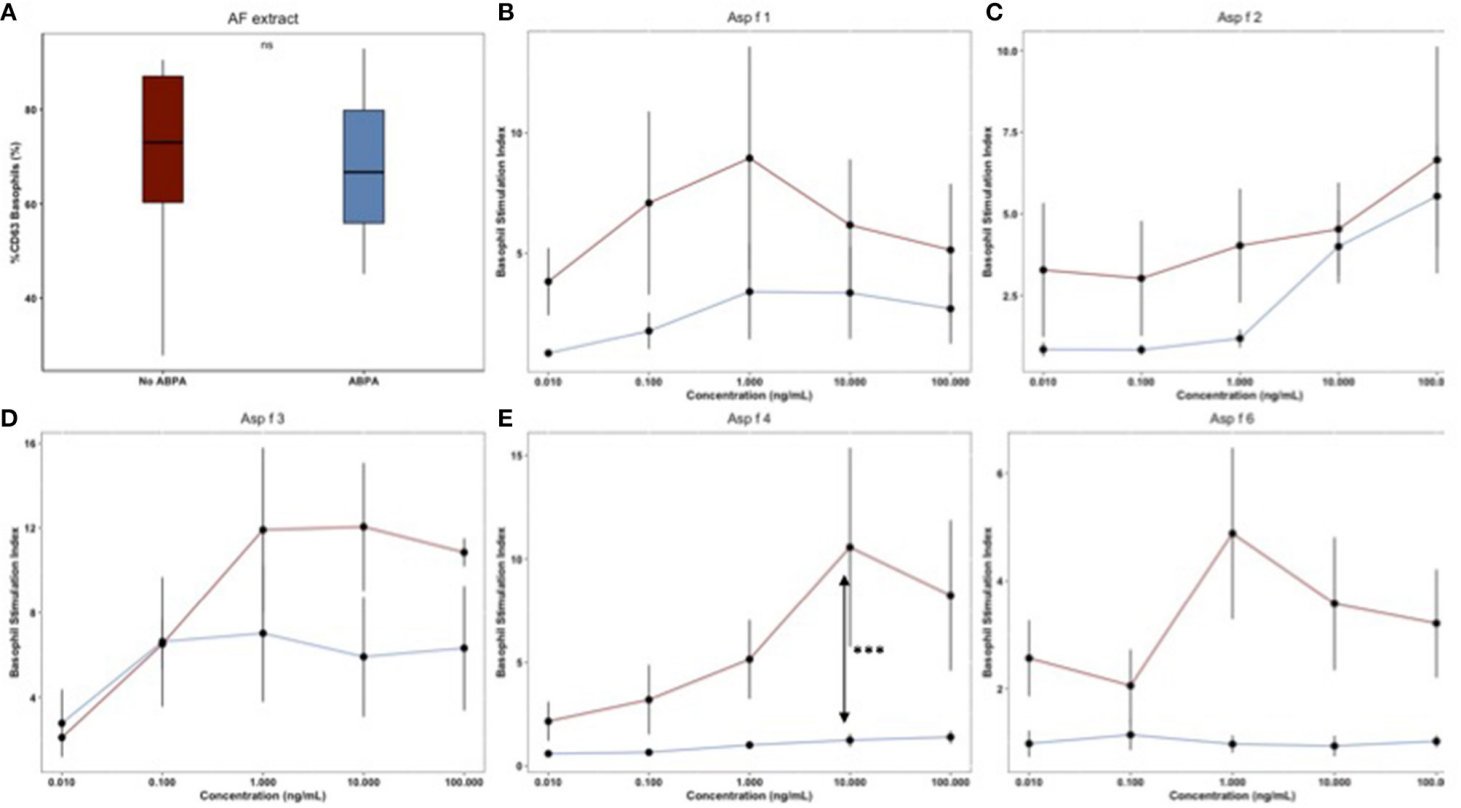
**(A–E)** Mean BAT responses for AF molecular components: Asp f 1, Asp f 2, Asp f 3, Asp f 4, Asp f 6. Mean (SD) responses for AF extract and each AF molecular component's concentration were calculated for ABPA (*n* = 6, red lines) and AF sensitized (*n* = 6, blue lines) patients. Significant difference was calculated at each point.

In positive (SI ≥ 2) BAT with AF extract, a dose-response BAT was performed with each of the five AF molecular components. Six AF-S and 6 ABPA patients were tested (Figure 3). There was no significant difference in BAT AF responses for these patients (Figure 3A). The mean responses with each AF molecular component were higher in ABPA patients, but only BAT with Asp f 4 at 10 ng/mL reached the significance level (basophil SI of 10.56 in ABPA group vs. 1.24 in no ABPA group, *p* = 0.0002) (Figures 3B–E).

### Matrix Correlation

**Figure 4:**
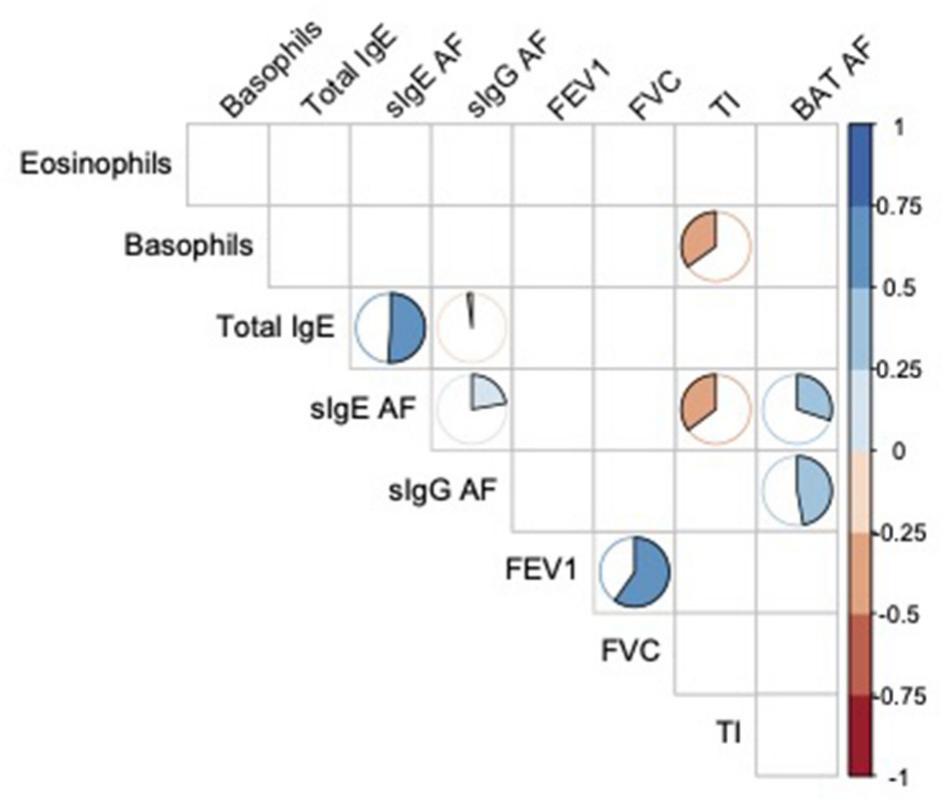
Spearman's correlation matrix of eosinophils and basophils count, total IgE, specific IgE and IgG AF levels, pulmonary function tests results and functional cellular assays. Only significant correlations (a = 0,05) are illustrated by a pie chart. The fill rate of pie chart is proportional to correlation coefficient. AF, Aspergillus fumigatus; FEV1, Forced expiratory volume in 1 sec; FVC, forced vital capacity; TI, Tiffeneau index.

As illustrated in Figure 4, total IgE were more strongly correlated with sIgE to AF (*r* = 0.53, *p* = 0.01) than IgG to AF (*r* = −0.03, *p* = 0.001), although sIgE were correlated with sIgG to AF (*r* = 0.29, *p* = 0.003). BAT AF was correlated with sIgG (*r* = 0.45, *p* < 10^−3^) and IgE (*r* = 0.35, *p* = 0.01) to AF. Tiffeneau index was inversely correlated with the basophil count (r = −0.36, *p* = 0.03) and sIgE to AF levels (*r* = −0.35, *p* = 0.01). The FEV1 (Forced Expiratory Volume in 1 second) was strongly correlated with FVC (Forced Vital Capacity) (*r* = 0.62, *p* = < 10^−3^).

## Discussion

Data on BAT as a tool for ABPA diagnosis are scarce, with only five previous studies from our and other teams (12–16). Four of them have focused on CF patients, an underlying disease associated with the highest ABPA incidence (25) and the global conclusion was that BAT could improve ABPA diagnosis in CF patients. The study of Prasad et al. displayed more contrasted results on asthmatic patients. The present study brings further insights, from a clinical viewpoint, into the usefulness of BAT for ABPA diagnosis in asthmatic patients.

First, we demonstrated the absence of a plateau phase in basophil responses to high AF concentrations. This finding suggests that the basophil signaling switch induced by supraoptimal allergen concentrations does occur with AF extract, similarly to experimental conditions of highest response induced by equivalent amounts of allergens and IgE bound to mast cell and basophil FcεRI (26, 27). The bell-shaped curve of AF-induced basophil activation indicates the need for standardized concentrations of AF extract for BAT, and its potential use as a follow-up test through the monitoring of CD-sens and EC-50 (28, 29).

Secondly, we confirmed that BAT with AF extract performs better than humoral markers (total IgE, IgE to AF and IgG to AF) for ABPA diagnosis. With a 100% negative predictive value, BAT could assist with ruling out ABPA in clinical settings.

In order to improve ABPA diagnostic accuracy, we performed BAT with AF molecular components in BAT AF-positive patients from our cohort. The soluble form of ImmunoCAP® antigens was employed. Overall, ABPA patients displayed higher BAT responses to all the molecular components as compared to AF crude extract. Asp f 4 BAT significantly discriminated ABPA from mere AF-sensitization. Serum sIgE to Asp f 4 has been reported as a better discriminant than AF extract and other AF molecular components for ABPA diagnosis in CF patients (9, 30–32). Hence, IgE immunization against Asp f 4 appears as a relevant marker of ABPA pathophysiology. Functions of this protein in *Aspergillus sp* have not been described yet. Ramachandran *et al* have studied the structure and immunogenicity of this protein, providing evidence of the key role of C-Terminal cysteine residues for IgE binding (33).

Basophil activation has been correlated with a strong and effective allergic immune activation, lung damage and pulmonary symptoms (34). We speculate that strong BAT responses in ABPA patients are related to their complex molecular sensitization profile (35).

The main limitations of our study were its monocentric design and the relatively small size of the cohort, hence calling for confirmation through larger studies. The main novelty of this study is the evidence of an improved discrimination between AF-S and ABPA, by using AF molecular components. In conclusion, cellular functional assays are easy to implement in the routine clinical laboratory for direct and personalized evaluation of each patient's functional responses to AF extract and proteins. They might thus be the next first-line test for ABPA diagnosis.

## Data Availability Statement

The raw data supporting the conclusions of this article will be made available by the authors, without undue reservation.

## Ethics Statement

Ethical review and approval was not required for the study on human participants in accordance with the local legislation and institutional requirements. Written informed consent for participation was not required for this study in accordance with the national legislation and the institutional requirements.

## Author Contributions

MM, MG, CG, and JV contributed conception and design of the study. MM, YS, and FM performed the experiments. CC, SR, and MR-G performed the statistical analysis. J-LM wrote the first draft of the manuscript. All authors contributed to manuscript revision and read and approved the submitted version.

## Funding

This study was supported by the Institut Hospitalo-Universitaire (IHU) Méditerranée Infection, the French National Research Agency under the program Investissements d'avenir, reference ANR-10-IAHU-03, and the Région Provence Alpes Côte d'Azur and European funding FEDER PRIMMI.

## Conflict of Interest

The authors declare that the research was conducted in the absence of any commercial or financial relationships that could be construed as a potential conflict of interest.

## Publisher's Note

All claims expressed in this article are solely those of the authors and do not necessarily represent those of their affiliated organizations, or those of the publisher, the editors and the reviewers. Any product that may be evaluated in this article, or claim that may be made by its manufacturer, is not guaranteed or endorsed by the publisher.

## Abbreviations

ABPA: Allergic Bronchopulmonary Aspergillosis
AF: *Aspergillus fumigatus*
AUC: Area under the curve
BAT: Basophil Activation Test
CF: Cystic fibrosis
COPD: Chronic Obstructive Pulmonary Disease
FcεR: Fc epsilon Receptor
fMLP: Formyl-methionyl-leucyl-phenylalanine
Ig: Immunoglobulin
ISHAM: International Society for Human & Animal Mycology
PHA: Phytohemagglutinin
SI: Stimulation Index.
