# Supplementary material for: Basophil Activation Test With Aspergillus Molecules: The Case for ABPA
Source: Front Allergy. 2022 Jun 22;3:898731. doi: 10.3389/falgy.2022.898731 (PMC9552950; doi:10.3389/falgy.2022.898731)
Supplement: Supplementary file 1 [file Data_Sheet_1.PDF]

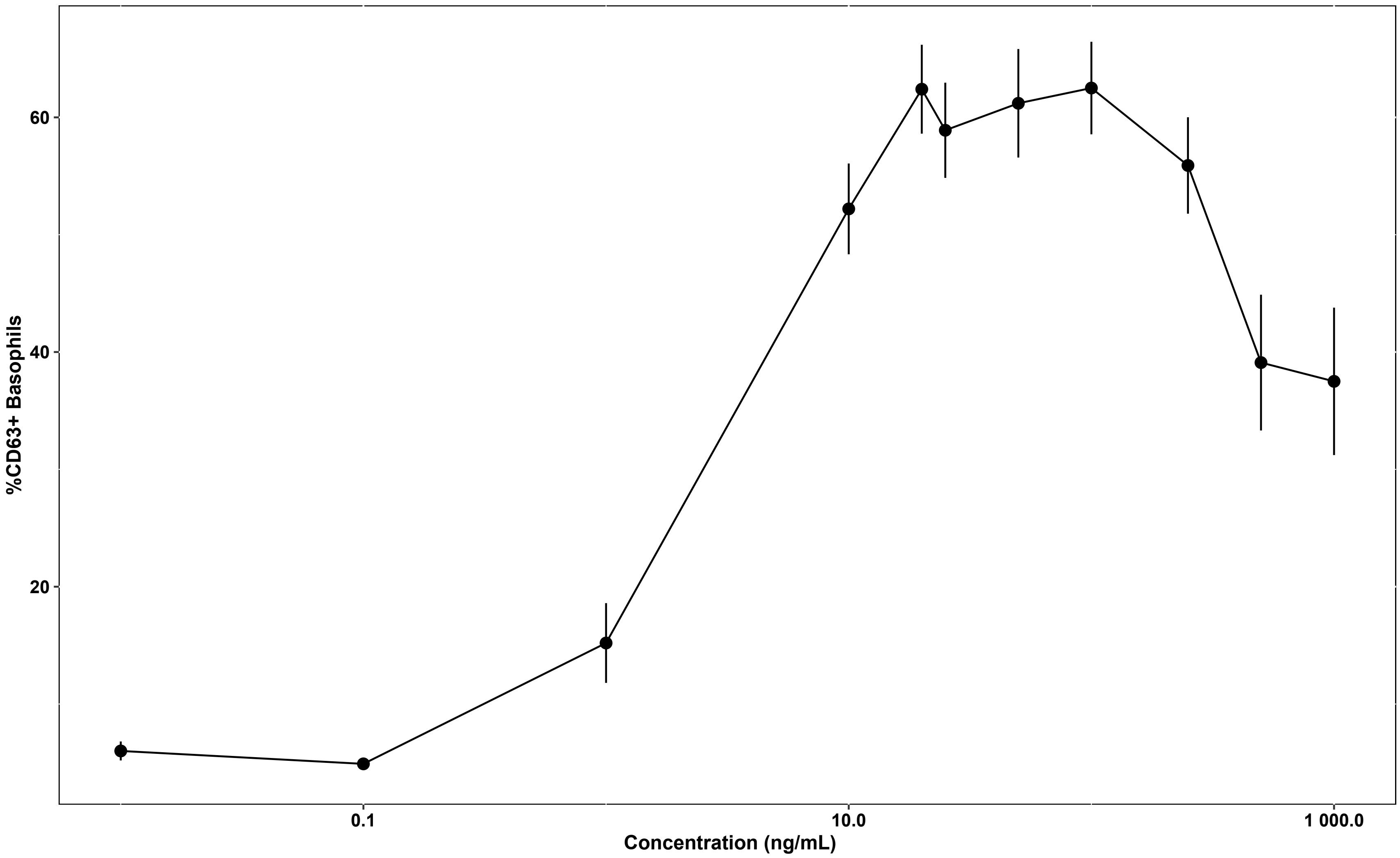

Sup Figure 1. Mean dose-response of basophil activation with *Aspergillus fumigatus* extract.
Dose-response of data from 3 patients with a positive basophil activation test to *Aspergillus fumigatus* extract, using the final concentrations of 0.01, 0.1, 1, 10, 20, 25, 50, 100, 250, 500 and 1,000 ng/mL.
